# Drivers of success: improving implementation research tools for better health outcomes

**DOI:** 10.1186/s12887-023-04471-7

**Published:** 2024-02-28

**Authors:** Quinhas Fernandes, Orvalho Augusto, Kenneth Sherr

**Affiliations:** 1 National Directorate of Public Health, Ministry of Health, Maputo, Mozambique; 2 Department of Global Health, University of Washington, Seattle, WA USA; 3 Community Health Department, School of Medicine, Eduardo Mondlane University, Maputo, Mozambique; 4 Department of Industrial & Systems Engineering, University of Washington, Seattle, WA USA; 5 Department of Epidemiology, University of Washington, Seattle, WA USA

**Keywords:** Under-five mortality, Exemplar countries, Implementation science, EPIAS, Low- and middle-income countries

## Abstract

A thorough examination of context, and how it influences implementation of evidence-based interventions, is a promising strategy for enhancing child survival initiatives. Spreading approaches that are identified as drivers of successful reduction in under-five mortality from ‘exemplar’ countries could be pivotal in leading to reductions in other settings facing stagnant mortality rates, in particular for low- and middle-income countries with high disease burden and insufficient programmatic capacity to effectively implement evidence-based interventions at scale. Yet there remains a lack of robust analytic methods to accurately assess mortality and describe the drivers of interventions’ implementation success at both national and subnational levels. The field of implementation science and its defining targets and tools is well positioned to address this knowledge gap by integrating qualitative and quantitative research methods into an adaptable evaluation framework that can be tailored to meet the specific needs across varying country contexts. These tools enhance the measurement of population health outcomes and provide crucial evidence on implementation barriers and facilitators that can inform policies that can be adjusted for diverse contexts. This commentary aims to emphasize the role of implementation research in understanding how exemplar countries achieved significant improvements in child survival and in identifying replicable lessons for other settings. Ultimately, all manuscripts underscore the relevance of implementation research in bolstering the reduction of under-five mortality.

## Background

Over the past three decades, remarkable progress has been achieved in addressing the leading causes of child deaths, resulting in a global decrease of over 50% in the under-five mortality rate. Worldwide, 62 countries met the Millennium Development Goal 4 target by 2015 [1]. Despite notable achievements at the country level, significant disparities persist across subnational regions. Insights gleaned from “exemplar” countries – those that have surpassed anticipated reductions in under-five mortality compared to similar countries in geography or socioeconomic conditions – may provide salient and actionable lessons. The lessons are critical to tracking existing disparities, optimizing the limited resources, and accelerating progress towards the Sustainable Development Goals.

The adoption of standardized implementation science frameworks and robust analytic designs, such as quasi-experiments and mixed methods inquiry, is increasing. These methodologies facilitate a comprehensive understanding of implementation processes and outcomes, encompassing the multi-level implementation determinants (barriers and facilitators). They enhance causal inference on country-level actions leading to under-five mortality reduction and complement results from trials that establish the efficacy of evidence-based interventions (EBIs) for preventing childhood mortality under controlled conditions [2]. The work described in this supplement provides evidence and essential guidance for low- and middle-income countries (LMICs) struggling to implement and scale-up EBIs to address under-five mortality. The supplement materials unpack how and why the six exemplar countries (Bangladesh, Ethiopia, Nepal, Peru, Rwanda, and Senegal) made such notable progress. This is a critical step to translating knowledge into practical application in other settings.

It is estimated that full and sustained coverage of EBIs delivered at or around the time of birth can lead to as much as a 72% reduction in neonatal mortality if implemented with high fidelity and quality [3]. Notably, available EBIs targeting the leading causes of under-five mortality have been adopted as routine policy across most LMICs, with numerous global financing mechanisms in place to ensure their accessibility [4]. Despite the integration of EBIs into normative guidelines, and greatly expanded access to EBIs at a national level, many LMICs have experienced slowdowns in the pace of under-five mortality reductions. This stagnation is due to a mix of unaddressed neonatal mortality, subnational disparities in EBI coverage, and insufficient guidance and challenges to translate knowledge into effective EBI delivery [5, 6]. Addressing these subnational disparities and ensuring high-fidelity and high-quality EBI delivery is essential to bend the curve on under-five mortality.

Contextual factors shape how well EBIs are delivered across settings, and therefore their relative impact on mortality. Studying these factors, including how they determine and change the delivery of EBIs, is critical to accomplishing the under-five mortality reduction desideratum [6]. Specifically, applying implementation research tools is imperative to scrutinize contextual factors that influence the delivery of EBIs. This approach builds on the body of knowledge on evidence-based strategies to strengthen health systems across settings. Ultimately, it seeks to enhance and sustain the delivery of EBIs, maximizing their potential impact on population-level health indices.

### A critical analysis of implementation research tools used to assess drivers of EBI implementation

Tools from the emerging field of implementation science – such as frameworks, implementation strategies, and dissemination approaches – can effectively target and bridge gaps in delivering priority EBIs. Furthermore, these tools aid in communicating outcomes to a broader audience [7]. Regardless of the approachability of specific frameworks, there is no unanimity on how to select the appropriate framework for a particular question, whether to use one or to combine multiple frameworks, or how to meaningfully integrate data across domains of interest [8]. Importantly, most implementation science frameworks lack validation in LMICs, limiting their reliability and usefulness. For example, a systematic review that assessed the application of EPIS (Exploration, Preparation, Implementation, Sustainment) framework identified only one study that used this framework in sub-Saharan Africa (South Africa) [9]. Moreover, there’s a lack of application of the Consolidated Framework for Implementation Research (CFIR) – another commonly used implementation science framework – within LMICs [10]. In this supplement, the investigators developed a hybrid framework based on existing frameworks (CFIR, EPIS, and RE-AIM) [11] to describe how EBIs were implemented and outcomes achieved, specifically in LMICs. As they note, the existing frameworks alone could not accurately cover relevant themes surfaced in investigating EBI implementation. Recognizing “adaptation” as a critical characteristic in the implementation and scale-up of EBIs, EPIS was expanded to EPIAS [12]. EPIAS was tested through a process of application to describe salient contextual factors and capture drivers of success from six countries with a greater reduction in under-five mortality. EPIAS efficiently mapped critical implementation contextual determinants, including the need to i) engage leaderships and communities; ii) adapt EBIs and strategies to the context; iii) embed EBIs within the existing health delivery systems; and iv) consider socioeconomic disparities. Despite providing a framework for identifying determinants of implementation success in LMIC settings, further inquiry is merited to assess how EPIAS applies in other contexts, including in countries with poorer performance.

The implementation research methods that were used to identify exemplar countries – which involved identifying countries that outperformed the average, followed by mixed methods enquiry to identify drivers of this positive deviance – would theoretically apply at a subnational level and could be used by country leadership to identify and spread what works in practice within a country’s borders. However, the field faces a number of constraints in carrying out this exercise. First, there remain substantial gaps in measuring under-five mortality (as well as other population-level health indices) in LMIC settings lacking robust and representative vital statistic systems. Gold standard population-level surveys are infrequently conducted, and imprecise in estimating mortality rates, particularly at subnational administrative levels. Second, while there are acceptable model-based approaches to estimate under-five mortality at the subnational level, they insufficiently capture important determinants of mortality, are not context-adjusted, and make strong assumptions that are often hard to hold. Given the intention to provide generalizable results to guide replication in other settings, caution is merited in causally attributing mortality improvements to specific contextual determinants or implementation strategies. Therefore, conceptually, implementation research methods applied in exemplar countries could define a model for providing granular evidence relevant for informing policies and adjustments to implementation efforts. To achieve this end, we believe it is pertinent to consolidate frameworks such as EPIAS for LMIC settings, in addition to improving population health measurement at subnational levels, as these estimates will define the sampling to study contextual drivers of differential performance.

The application of mixed methods has gained relevance in implementation research, particularly by maximizing synergies between quantitative and qualitative methods to measure the impact of EBIs and simultaneously explain how, why, and in what context EBIs work. Effective mixed methods inquiry is not just applying both quantitative and qualitative research techniques, but intentionally and consistently integrating quantitative and qualitative results to increase the relevance and rigor of study evaluations and findings [13]. The presented mixed methods case studies used quantitative data to identify how exemplar countries made progress in reducing under-five mortality. Further specifying operative details on how qualitative and quantitative data were integrated, including clarity on the reason for applying a mixed methods design and how both data types informed each other in every step of the study facilitates study interpretation and provides a model for others to replicate. Explaining how quantitative data informed the qualitative sampling strategy and data collection, as well as how findings were meaningfully combined and interpreted, is often neglected in mixed methods inquiry, thereby distorting mixed methods’ overall goal.

## Conclusion

The work presented in this supplement is encouraging from several perspectives. It sets a stage for implementation researchers, particularly those in LMICs, to improve evaluations of what works to increase coverage of EBIs and reduce under-five mortality. It stands as a valuable demonstration of a harmonized methodology tailored for intricate evaluations spanning multiple countries. This approach yields systematic outcomes that hold the potential for greater generalizability, accelerating mortality reductions in high-need settings. It helps to understand policy implementation processes and outcomes as well as stakeholder dynamics in LMICs. While we focused on child mortality, we firmly believe that EPIAS is sufficiently flexible to be applied to other health areas and across implementation strategies. As a framework adapted specifically for LMICs, EPIAS should be tested by other implementation researchers and funders to advance the science of implementation, and bolster efforts to achieve the Sustainable Development Goals.

## Data Availability

Not Applicable (No data were generated during the study).

## Abbreviations

CFIR: Consolidated Framework for Implementation Research
EBI: Evidence-based interventions
EPIS: Exploration, Preparation, Implementation, Sustainment framework
EPIAS: Exploration, Preparation, Implementation, Adaptation, Sustainment framework
LMIC: low- and middle-income countries
RE-AIM: Reach, Effectiveness, Adoption, Implementation, Maintenance
U5MR: under-five mortality rate

## References

[1] You D, Hug L, Ejdemyr S, Idele P, Hogan D, Mathers C (2015). Global, regional, and national levels and trends in under-5 mortality between 1990 and 2015, with scenario-based projections to 2030: a systematic analysis by the UN Inter-agency Group for Child Mortality Estimation. The Lancet..

[2] Geldsetzer P, Fawzi W (2017). Quasi-experimental study designs series—paper 2: complementary approaches to advancing global health knowledge. J Clinic Epidemiol..

[3] Darmstadt GL, Bhutta ZA, Cousens S, Adam T, Walker N, de Bernis L (2005). Evidence-based, cost-effective interventions: how many newborn babies can we save?. The Lancet..

[4] Lassi ZS, Bhutta ZA. Community-based intervention packages for reducing maternal and neonatal morbidity and mortality and improving neonatal outcomes. Cochrane Datab Syst Rev. 2015 Mar;2015(3) 10.1002/14651858.CD007754.pub3 PMC8498021 25803792

[5] Dwyer-Lindgren L, Squires ER, Teeple S, Ikilezi G, Allen Roberts D, Colombara D (2018). Small area estimation of under-5 mortality in Bangladesh, Cameroon, Chad, Mozambique, Uganda, and Zambia using spatially misaligned data. Popul Health Metrics..

[6] Fernandes QF, Wagenaar BH, Anselmi L, Pfeiffer J, Gloyd S, Sherr K (2014). Effects of health-system strengthening on under-5, infant, and neonatal mortality: 11-year provincial-level time-series analyses in Mozambique. The Lancet Global Health..

[7] Kirk MA, Kelley C, Yankey N, Birken SA, Abadie B, Damschroder L (2016). A systematic review of the use of the Consolidated Framework for Implementation Research. Implement Sci..

[8] Birken SA, Powell BJ, Presseau J, Kirk MA, Lorencatto F, Gould NJ, et al. Combined use of the Consolidated Framework for Implementation Research (CFIR) and the Theoretical Domains Framework (TDF): a systematic review. Implement Sci : IS. 2017 Jan;12(1) 10.1186/s13012-016-0534-z PMC5217749 28057049

[9] Moullin JC, Dickson KS, Stadnick NA, Rabin B, Aarons GA (2019). Systematic review of the Exploration, Preparation, Implementation, Sustainment (EPIS) framework. Implement Sci..

[10] Means AR, Kemp CG, Gwayi-Chore MC, Gimbel S, Soi C, Sherr K (2020). Evaluating and optimizing the consolidated framework for implementation research (CFIR) for use in low- And middle-income countries: A systematic review. Implement Sci..

[11] Damschroder LJ, Aron DC, Keith RE, Kirsh SR, Alexander JA, Lowery JC (2009). Fostering implementation of health services research findings into practice: A consolidated framework for advancing implementation science. Implement Sci..

[12] Hirschhorn LR, Frisch M, Ntawukuriryayo JT, VanderZanden A, Donahoe K, Mathewos K (2021). Development and application of a hybrid implementation research framework to understand success in reducing under-5 mortality in Rwanda. Gates Open Res..

[13] Sandelowski M (2000). Combining qualitative and quantitative sampling, data collection, and analysis techniques in mixed-method studies. Res Nursing Health..

